# Genome-wide association study of primary open-angle glaucoma in continental and admixed African populations

**DOI:** 10.1007/s00439-018-1943-7

**Published:** 2018-10-13

**Authors:** Pieter W. M. Bonnemaijer, Adriana I. Iglesias, Girish N. Nadkarni, Anna J. Sanyiwa, Hassan G. Hassan, Colin Cook, GIGA Study Group, Mark Simcoe, Kent D. Taylor, Claudia Schurmann, Gillian M. Belbin, Eimear E. Kenny, Erwin P. Bottinger, Suzanne van de Laar, Susan E. I. Wiliams, Stephen K. Akafo, Adeyinka O. Ashaye, Linda M. Zangwill, Christopher A. Girkin, Maggie C. Y. Ng, Jerome I. Rotter, Robert N. Weinreb, Zheng Li, R. Rand Allingham, Eyes of Africa Genetics Consortium, Abhishek Nag, Pirro G. Hysi, Magda A. Meester-Smoor, Janey L Wiggs, NEIGHBORHOOD Consortium, Michael A. Hauser, Christopher J. Hammond, Hans G. Lemij, Ruth J. F. Loos, Cornelia M. van Duijn, Alberta A. H. J. Thiadens, Caroline C. W. Klaver

**Affiliations:** 1 Department of Ophthalmology, Erasmus MC, Rotterdam, The Netherlands; 2 Department of Epidemiology, Erasmus MC, Rotterdam, The Netherlands; 3 The Rotterdam Eye Hospital, Rotterdam, The Netherlands; 4 Department of Clinical Genetics, Erasmus MC, Rotterdam, The Netherlands; 5 The Charles Bronfman Institute of Personalized Medicine, Icahn School of Medicine at Mount Sinai, New York, NY, USA; 6 Division of Nephrology, Department of Medicine, Icahn School of Medicine at Mount Sinai, New York, USA; 7 Department of Ophthalmology, Muhimbili University of Health and Allied Sciences/Muhimbili National Hospital, Dar es Salaam, Tanzania; 8 Department of Ophthalmology, Comprehensive Community Based Rehabilitation in Tanzania (CCBRT) Hospital, Dar es Salaam, Tanzania; 9 Division of Ophthalmology, University of Cape Town, Cape Town, South Africa; 10 Department of Twin Research and Genetic Epidemiology, King’s College London, London, UK; 11 Department of Pediatrics, The Institute for Translational Genomics and Population Sciences, Los Angeles Biomedical Research Institute, Harbor-UCLA Medical Center, Torrance, CA, USA; 12 Genetic and Genomic Sciences, Icahn School of Medicine at Mount Sinai, New York, NY, USA; 13 The Center for Statistical Genetics, Icahn School of Medicine at Mount Sinai, New York, NY, USA; 14 The Institute for Genomics and Multiscale Biology, Icahn School of Medicine at Mount Sinai, New York, NY, USA; 15 Department of Ophthalmology, University Medical Center, Utrecht, The Netherlands; 16 Division of Ophthalmology, Department of Neurosciences, University of the Witwatersrand, Johannesburg, South Africa; 17 Unit of Ophthalmology, Department of Surgery, University of Ghana School of Medicine and Dentistry, Accra, Ghana; 18 Department of Ophthalmology, College of Medicine, University of Ibadan, Ibadan, Nigeria; 19 Department of Ophthalmology, Hamilton Glaucoma Center, Shiley Eye Institute, University of California San Diego, La Jolla, CA, USA; 20 Department of Ophthalmology, University of Alabama at Birmingham School of Medicine, Birmingham, AL, USA; 21 Department of Biochemistry, Center for Diabetes Research, Wake Forest School of Medicine, Winston-Salem, NC, USA; 22 Genome Institute of Singapore, Singapore, Singapore; 23 Department of Ophthalmology, Duke University, Durham, NC, USA; 24 Department of Ophthalmology, Harvard Medical School, Boston, MA, USA; 25 Department of Medicine, Duke University Medical Center, Durham, NC, USA; 26 Glaucoma Service, The Rotterdam Eye Hospital, Rotterdam, The Netherlands; 27 The Mindich Child Health and Development Institute, Icahn School of Medicine at Mount Sinai, New York, NY, USA; 28 Department of Ophthalmology, Radboud University Medical Center, Nijmegen, The Netherlands

## Abstract

Primary open angle glaucoma (POAG) is a complex disease with a major genetic contribution. Its prevalence varies greatly among ethnic groups, and is up to five times more frequent in black African populations compared to Europeans. So far, worldwide efforts to elucidate the genetic complexity of POAG in African populations has been limited. We conducted a genome-wide association study in 1113 POAG cases and 1826 controls from Tanzanian, South African and African American study samples. Apart from confirming evidence of association at *TXNRD2* (rs16984299; OR_[T]_ 1.20; *P* = 0.003), we found that a genetic risk score combining the effects of the 15 previously reported POAG loci was significantly associated with POAG in our samples (OR 1.56; 95% CI 1.26–1.93; *P* = 4.79 × 10^−5^). By genome-wide association testing we identified a novel candidate locus, rs141186647, harboring *EXOC4* (OR_[A]_ 0.48; *P* = 3.75 × 10^−8^), a gene transcribing a component of the exocyst complex involved in vesicle transport. The low frequency and high degree of genetic heterogeneity at this region hampered validation of this finding in predominantly West-African replication sets. Our results suggest that established genetic risk factors play a role in African POAG, however, they do not explain the higher disease load. The high heterogeneity within Africans remains a challenge to identify the genetic commonalities for POAG in this ethnicity, and demands studies of extremely large size.

## Introduction

Glaucoma is the leading cause of irreversible blindness worldwide (Tham et al. 2014). The disease is an optic neuropathy characterized by loss of retinal ganglion cells resulting in peripheral visual field defects. Later in the disease process, the visual field defects may involve central vision leading to blindness. Primary open-angle glaucoma (POAG) is the commonest subtype of glaucoma. Intraocular pressure (IOP), family history, age, and ancestry are established risk factors. In particular persons of African ancestry have 3–5 × increased risk of POAG, and have a more severe course of disease with a higher risk of blindness (Cook 2009; Kyari et al. 2013). This ethnic predilection along with the familial nature strongly suggests that genetic factors contribute to the pathogenesis of POAG.

Recently, progress has been made in the identification of associated variants using linkage analysis and genome-wide association studies (GWAS). Rare variants with large effects have been identified in *MYOC* and *OPTN*, common variants with smaller effect have been reported in genomic regions that include *CAV1-CAV2, CDC7-TGFRB3, TMCO1, CDKN2B-AS1, ABCA1, AFAP1, GMDS, TXNRD2, ATXN2, FOXC1, GAS7, ARHGEF12, SIX6*, 8q22 and *PMM2* (Bailey et al. 2016; Burdon et al. 2011; Chen et al. 2014; Liu et al. 2013; Hysi et al. 2014; Li et al. 2015; Springelkamp et al. 2015; Thorleifsson et al. 2010; Wiggs et al. 2012). However, these loci explain only 5–10% of cases, leaving the heritability of POAG largely unexplained. Most genetic studies were predominantly conducted in European and Asian populations, leaving African ancestry underrepresented up to now. Recent studies in Africans or in cohorts of African descent (i.e., Ghana, South Africa and in African Americans) could not replicate most of the loci previously identified in GWAS of European and Asian populations (Cao et al. 2012; Liu et al. 2013; Williams et al. 2015).

Gene finding can be more effective in study populations where the disease is more common, of earlier onset and more severe. Therefore, in this study, we conducted a genome-wide meta-analysis using African black and South African colored POAG cases and controls, from the Genetics In Glaucoma patients from African descent study (GIGA) recruited at hospitals from South Africa and Tanzania and African Americans enrolled in the BioMe (2018) biobank.

## Results

The GIGA dataset consisted of 444 participants from South Africa (*N*_POAG_ = 297; *N*_control_ = 147) and 695 participants from Tanzania (*N*_POAG_ = 366; *N*_control_ = 329). The Tanzanian participants were all from the black African origin, 38% of South African participants were also from black African origin while the remaining 62% were self-reported South African Coloured (European, African, Asian admixed). The BioMe dataset consisted of POAG cases (*N* = 450) and controls (*N* = 1350) and were all African American. The clinical and demographic characteristics of the GIGA and BioMe participants have been summarized in Table 1.

### Association of previously reported POAG loci in African populations

First, we tested the association of the 15 previously established POAG SNPs identified in GWAS of European and Asian populations in the GIGA and BioMe datasets (Bailey et al. 2016; Burdon et al. 2011; Chen et al. 2014; Liu et al. 2013; Hysi et al. 2014; Li et al. 2015; Springelkamp et al. 2015; Thorleifsson et al. 2010; Wiggs et al. 2012). None of these SNPs replicated at a nominal significance level (*P* < 0.05) in any single ethnicity (Supplementary Table 1), nor in a combined analysis (Table 2 exact replication). Because linkage disequilibrium (LD) patterns may differ between the study populations of the reported GWAS and the current African study participants, we also searched for evidence of transferability of the SNPs. Locuszoom plots were made using the LD pattern of Europeans and Asians (1000Genomes) to investigate whether SNPs in high LD (*r*^2^ > 0.8) with the original lead SNP showed evidence of association in our study (Supplementary Fig. 1). This “local” replication strategy queried a 500 kb window centered on the lead SNP, and yielded a total of 246 SNPs in LD (*r*^2^ > 0.8) with the 15 lead SNPs. Of these 246 SNPs, three SNPs in the *TXNRD2, CDKN2B-AS1*, and *TMCO1* loci were significantly associated with POAG in our study (*P* < 0.05) (Table 2, local replication), rs16984299 in *TXNRD2* had similar effect size as reported by Cook Bailey et al.(Bailey et al. 2016) and survived multiple testing (OR_[T]_ 1.20; 95% CI 1.06–1.35; *P*_Bonferonni_ = 0.049) when the association was corrected for the effective number of SNPs (*N* = 16) in the queried 500 kb window. In addition, we also analyzed three independent POAG variants found in African Americans from the Women Health Initiative (Hoffmann et al. 2014). We found rs192917960 at the *RBFOX1* locus associated with POAG in BioMe (*P* = 0.02, Supplementary Table 2), but this association did not withstand correction for multiple testing.

Next, we compared effect sizes from the combined analysis of GIGA and BioMe with the effect sizes from published GWAS reports. In total, 12 out of the 15 known lead SNPs had a consistent direction of effect (Supplementary Fig. 2). Allele frequencies for most SNPs were very similar in South African blacks, Tanzanian, and African American datasets, but markedly different compared with the European and Asian studies (Supplementary Fig. 3). In eight of the 15 SNPs, the effect allele had a considerable higher frequency in Africans while five were clearly less frequent compared to Europeans.

**Fig. 1:**
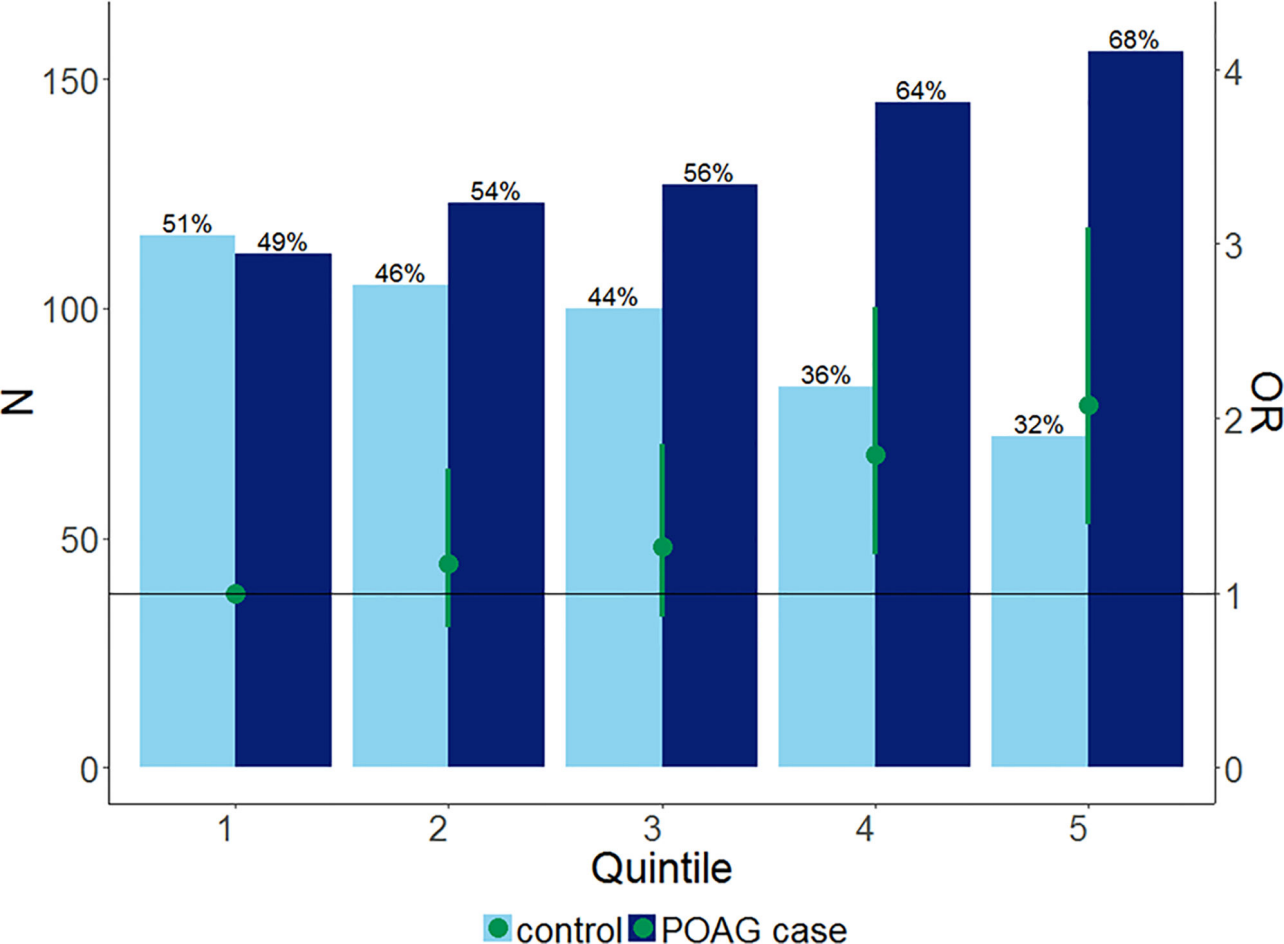
Genetic Risk Score. Genetic risk score based on the 15 known POAG-loci identified in Europeans and Asians GWAS (rs1192415, rs28504591, rs4619890, rs2745572, rs11969985, rs4236601, rs284489, rs10712703, rs2472493, rs58073046, rs7137828, rs10483727, rs3785176, rs9897123, rs16984299). Participants were grouped into quintiles of the genetic risk scores. Green circles represent the POAG odds ratio (adjusted for age, sex and principal components) when comparing each quintile to the lowest quintile (Q1 = reference line). The green-capped lines represent 95% CI of the POAG odds ratios. Bars represent the percentage of POAG cases (dark blue) and controls (light blue) per quintile.

To study the contribution of the known SNPs to the risk of POAG in GIGA, we calculated a multilocus Genetic Risk Score (GRS) based on 15 known SNPs. Three known SNPs for *TXNRD2, CDKN2B-AS1*, and *TMCO1* were replaced by the proxies that were identified by the local replication approach described above. Scores were weighted based on the effect sizes found in the GWAS meta-analysis of European populations. The GRS, adjusted for age, sex, and first five principal components were associated with POAG in the GIGA sample (OR 1.56; 95% CI 1.26–1.93; *P* = 4.85 × 10^−5^). We then stratified the GRS in quintiles, and estimated the risk of POAG for each quintile relative to the lowest one (Fig. 1). Trend analysis showed a significant stepwise increase in the risk of POAG per quintile (*P*_trend_ = 2.81 × 10^−5^), with a twofold increase in POAG risk for the highest quintile compared to the lowest. The risk attributed to genetics was calculated in reference to the mean genetic risk score in the controls. We found that these 15 known variants taken all together attributed 4% (95% CI 2–6%) to the overall POAG risk in this study population when we adjusted for age, sex and principal components.

### Discovery (stage 1)

**Fig. 2:**
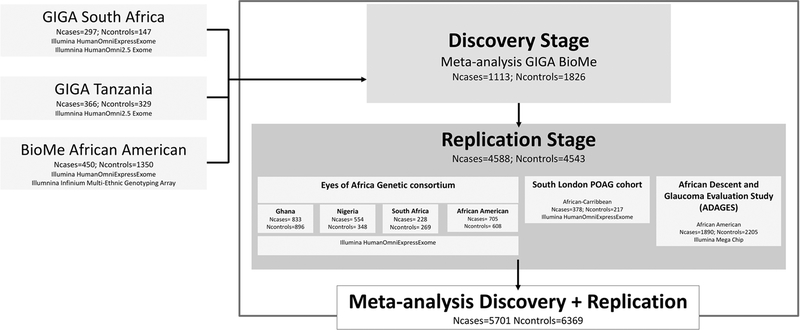
Study design

**Fig. 3:**
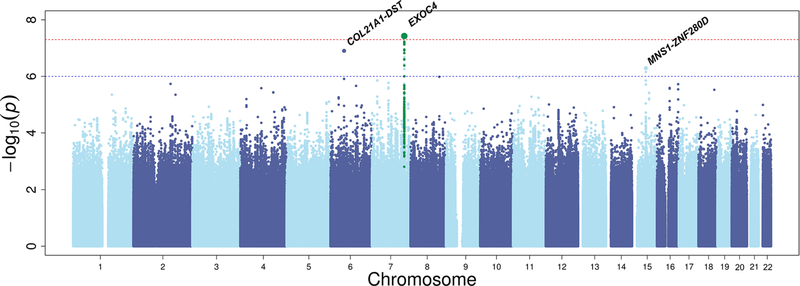
Manhattan plot for the association of genome-wide SNPs with primary open-angle glaucoma in GIGA BioMe meta-analysis. Manhattan plot of the GWAS meta-analysis of GIGA and BioMe (*N*=1113cases/*N* = 1826 controls). The figure shows −log10-transformed *P* values for all SNPs. The upper dotted horizontal line represents the genome-wide significance threshold of *P* < 5.0 × 10^−8^; the lower dotted line indicates a *P* value of 1 × 10^−6^. Green dots represents variants in that are in linkage disequilibrium (*r*^2^ > 0.6 1000 Genomes African ancestry) with the top SNP rs141186647.

**Fig. 4:**
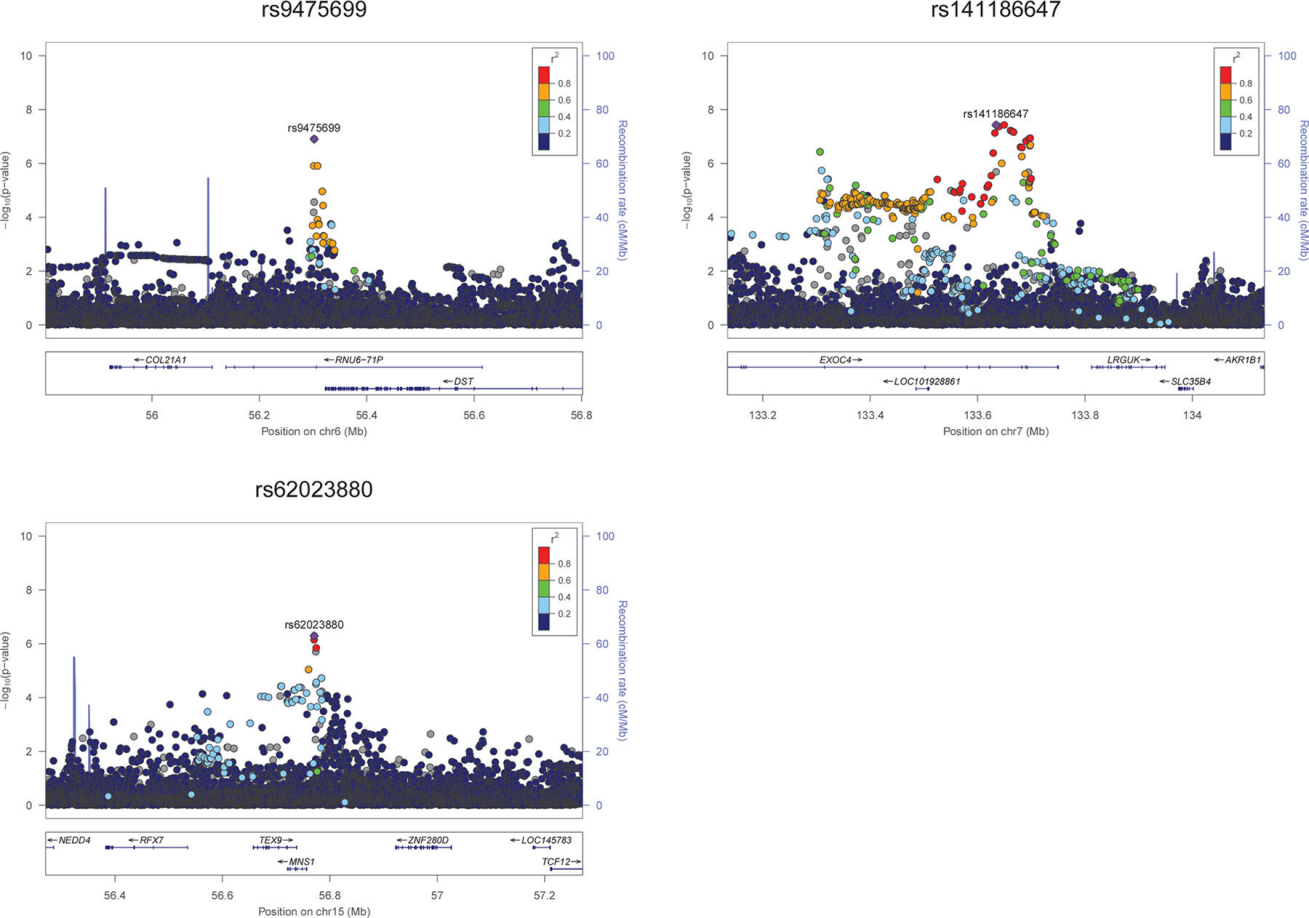
Regional Plots for SNPs *P <* 1 × 10^−6^ in the discovery stage (stage 1)

To identify new loci associated with POAG in African populations, we performed GWAS using our African ancestry datasets. The scheme of the study design is depicted in Fig. 2. In the discovery stage, we meta-analyzed GWAS results from the GIGA study (South Africa and Tanzania) and BioMe (African American) including in total 1113 POAG cases and 1826 controls. A total of 13.8 million SNPs were available after applying our QC and filtering criteria (see “Methods” section). The genomic inflation factor was 0.94 (SE 1.49 × 10^−6^) and the quantile–quantile plot did not show any systemic inflation in the association results, suggesting that confounding by cryptic population stratification was unlikely (Supplementary Fig. 4). The discovery association results across the whole genome are shown in Fig. 3. We identified one novel region reaching genome-wide significance (*P* < 5 × 10^−8^) in the discovery stage, and two suggestive regions (*P* < 1 × 10^−6^) (Table 3). The top newly associated SNPs were rs141186647[A] an intronic variant in *EXOC4* on chromosome 7 (OR 0.48; *P* = 3.15 × 10^−8^), rs9475699[A] downstream of *DST* on chromosome 6 (OR 1.65; *P* = 1.25 × 10^−7^), and rs62023880[A] upstream of *MNS1* on chromosome 15 (OR 1.39; *P* = 5.12 × 10^−7^). The regional association plots for these three SNPs are shown in Fig. 4. We did not observe any significant heterogeneity for these SNPs in the meta-analysis of GIGA and BioMe. The association results per ethnic group are provided in Supplementary Table 2, showing similar effects in Tanzanians, South Africans, and African Americans. Conditional and joint analyses did not identify any additional independent signals within the set of SNPs reaching *P* < 1 × 10^−6^. Additionally, we explored if haplotypes encompassing any of the three top SNPs were associated with POAG in GIGA BioMe, the results for this haplotype association analysis are provided in Supplementary Table 3.

### Replication of associated variants in African populations (stage 2 and stage 3)

All SNPs reaching *P <* 1 × 10^−6^ in stage 1 were followed-up in a replication (stage 2) comprising four independent African ancestral studies from South Africa, Ghana, Nigeria and African Americans (Eyes of Africa Genetic Consortium; *N*_cases_ = 2320; *N*_controls_ = 2121), the South London POAG case–control cohort comprising individuals from West African origin (*N*_cases_ = 378; *N*_controls_ = 217) and The African Descent and Glaucoma Evaluation Study (ADAGES) including African Americans (*N*_cases_ = 1890; *N*_controls_ = 2205). In total, 22 SNPs at the three independent loci were brought forward for replication. Variant rs9475699 (downstream of *DST*) reached a nominal level of statistical significance (OR 1.19, *P* = 0.032) in the Ghanaian study population (Supplementary Table 4). We then performed a meta-analysis of all six replication datasets (stage 2), first using a fixed effects model, and found no statistical significant replication (Table 4). Subsequent meta-analysis by means of the Han and Eskin random-effects model for SNPs with significant (*P* < 0.05) heterogeneity, also did not identify any SNPs with significant association. In stage 3, we performed a meta-analysis of all studies (stage 1 + stage 2), totaling 5701 POAG cases and 6369 controls. Given the high degree of heterogeneity observed in the fixed effect meta-analysis at this stage, we performed Han and Eskin random-effects model. Neither fixed effects nor Han and Eskin random-effect meta-analysis resulted in genome-wide significant signals (Table 4, Supplementary Table 4 and Supplementary Fig. 5).

### Cross-ethnic validation

We further investigated to what extent loci found in our African ancestry GWAS confer a risk of POAG in Europeans. We investigated the top three ranked loci from the discovery stage in two independent European ancestry studies from the National Eye Institute Glaucoma Human Genetics Collaboration (NEIGHBOR) and the Massachusetts Eye and Ear Infirmary (MEEI) (totaling 2606 POAG cases and 2606 controls) with imputed genotype data using the Haplotype Reference Consortium (HRC) (McCarthy et al. 2016). rs141186647 (*EXOC4*) and rs9475699 (*COL21A1-DST*) are rare in the European cohorts but had excellent imputation scores (MAF 0.00019, *r*^2^ 0.987; and MAF 0.0023, *r*^2^ 0.963, respectively). However, neither SNP demonstrated significant association in the European datasets (rs141186647 OR_[A]_ 5.09; *P* = 039; rs9475699 OR_[A]_ 1.27; *P* = 0.59). SNP rs62023880 (*MNS1-ZNF280D*) on chromosome 15, which was a common variant in the NEIGHBOR/MEEI sample (MAF = 0.15,) also did not show statistically significant replication (OR 0.947; *P* = 0.34).

### Bioinformatical lookup of functional and regulatory effects and expression of POAG-associated SNPs

We explored the functional and regulatory annotations of the three lead SNPs found in the discovery stage, including proxy-SNPs within high LD (*r*^2^ > 0.8 in 1000G AFR). The significant top hit rs141186647 at 7q33 represented an intronic variant within the Exocyst Complex Component 4 gene (*EXOC4*). The locus contains a set of SNPs in high LD that reside within the introns and within exon 15 (rs34608222; synonymous) of *EXOC4* of which only rs79198429 (*r*^2^ = 0.92 with rs141186647) is annotated as possibly disrupting; transcription factor binding (Regu-lomeDB score 3a; Supplementary Table 5). This variant is located inside a region annotated as an enhancer histone mark in multiple tissues by the RoadMap Epigenomics project, which is predicted to bind the transcriptional coactivator protein P300, and to alter five binding motifs including AP-1 transcription factor (Roadmap Epigenomics et al. 2015). None of the explored SNPs in this region were associated with eQTL’s.

In silico analyses of SNPs correlated with rs9475699 located 21 kb downstream the *DST* gene and rs62023880 neighboring *MNS1* gene did not identify any markers with evidence for gene regulatory effects.

To assess the expression of the annotated genes in human eye tissues, we examined the online Ocular Tissue Database (https://genome.uiowa.edu/otdb/) (Wagner et al. 2013). Expression of *EXOC4, DST* and *MNS1* was observed in tissues relevant to POAG, such as the trabecular meshwork, optic nerve head and optic nerve. Supplementary Table 6 depicts the differences in expression levels of these three genes across tissue types. In the optic nerve head, the highest level of expression was found for *DST* gene (PLIER 632.5).

### Gene-based tests

We performed gene-based tests using VEGAS2 (2018) on the GIGA BioMe meta-analysis results, and first investigated the 15 known POAG genes. None of these were significant at a nominal statistical level, the smallest *P* value was found for *FOXC1* (*P* = 0.103, nSNPs = 573) (Supplementary Table 7). We subsequently explored the gene-based test results of a total of 25,590 autosomal genes, using a Bonferroni corrected gene-based significance threshold of *P*_gene–based_< 1.95 × 10^−6^ (0.05/25590). The EXOC4 gene (*P* = 3.10 × 10^−5^) did not withstand Bonferroni correction.

## Discussion

To date, only European and Asian ancestry GWA studies have contributed to the 15 currently known genetic loci for POAG. Although the frequency of POAG in persons from African descent is high compared to those of European or Asian descent, studies of individuals of African descent are missing so far. The current study focuses on filling this gap. In this case–control study consisting of Africans from the African continent as well as of African Americans, we confirmed three POAG loci (*CDKN2B-AS1, TMCO1, TXNRD2*) at nominal significance that were previously found in Europeans, and report one novel candidate locus (*EXOC4*). A variant (rs1063192) near *CDKNB2-AS1* has previously been shown in the Afro-Caribbean population of Barbados, although this study could not replicate other known putative loci (Cao et al. 2012). Another insight gained from the current study was that the “local approach” rather than exact replication yielded these replicable findings in Africans. Interestingly, these proxy SNPs in Africans have a very similar effect size compared to the lead SNP in European GWAS.

This study has strengths and limitations. Of particular strength was the Pan-African origin of the study participants. Previous studies from the African continent were smaller and they all focused mainly on West Africans. Our study is the first genetic analysis which included East Africans. A probable disadvantage of applying a Pan-African approach must also be considered. The high genetic diversity present across African populations, even when they are geographically close, may reduce the likelihood of reproducing associations in multi-center studies. Other strengths were the careful diagnosis of cases, the strict criteria for controls, and the application of local replication. Optic discs were graded in an objective manner from fundus photographs by glaucoma experts using internationally accepted standards (Foster et al. 2002). Controls underwent the same review process as cases and had to be over 50 years of age to increase the diagnostic certainty of non-disease status. The limitations of our study include the relatively low power to detect genome-wide significance for small effect sizes, as reflected by the genomic inflation factor < 1.0, and the lack of a replication set from East Africa.

As the genome of African populations is much older, genetic diversity is increased, and LD across loci is decreased. Rather than focusing only on the lead SNPs from European/Asian GWAS, we considered all variants that were in strong European/Asian LD with the lead SNPs. We analyzed these variants in our African samples, and found evidence for nominal replication of three SNPs in *TMCO1* (rs28504591), *CDKN2B-AS1* (rs10712703), and *TXNRD2* (rs16984299), of which the latter withstood Bonferroni correction for the number of effective SNPs. The most significant SNP identified in GWAS is often not the causal variant (McCarthy and Hirschhorn 2008). We found similar effect sizes compared to the European GWAS for the three SNPs identified by the local replication approach. The overall weaker LD structure in Africans favors proximity of these proxy SNPs to the true causal variant. This makes it more likely that these proxies are functional. We, therefore, recommend candidate gene studies in African populations that failed to replicate known disease loci found in European or Asian populations to use this local approach.

Although this study found evidence that at least one known POAG gene plays a role in African glaucoma, we could not significantly replicate the remaining 14 associated SNPs even when we applied the local approach. Yet our GRS that was based on known European and Asian POAG SNPs showed a significant trend (*P* = 2.81 × 10^−5^) and a twofold increase in POAG risk comparing extreme risk groups. Of note, the allele frequency distributions for these SNPs differed markedly between our African study and the original European/Asian studies. This points towards differences in genetic architecture, and makes it difficult to estimate statistical power.

This study identified a novel candidate variant within the *EXOC4* gene in the meta-analysis of GIGA and BioMe. Recent reports provide evidence that this gene is implicated in cognitive traits as intelligence and educational attainment, and is also associated with the neurodegenerative Alzheimer’s disease (Okbay et al. 2016; Sherva et al. 2014; Sniekers et al. 2017). The *EXOC4* gene is ubiquitously expressed, and is particularly abundant in the brain. *EXOC4* encodes the SEC-8 protein, a component of a complex which is essential for exocytosis; it directs Golgi-derived secretory vesicles to specific docking sites on the plasma membrane. Exocyst proteins are needed for rapid membrane expansion, which happens during outgrowth of neurons and synaptogenesis. So far, the exocyst complex has not been studied in connection with glaucomatous optic neuropathy, however, it is expressed in the trabecular meshwork. In this tissue, it plays a role in the formation of invadopodia, protrusions that are important for releasing matrix metalloproteinase into the extracellular matrix to decrease trabecular outflow resistance (Han et al. 2013). Strikingly, our African POAG cases had high IOP, and it is intriguing to speculate that *EXOC4* contributes to POAG by interfering with matrix metalloproteinase release and trabecular outflow.

Replication of our genome-wide significant finding from the discovery set in our other African studies was challenging for this relatively rare variant. Meta-analysis of the discovery and replication stage showed considerable variation in effect size and direction of effect between the discovery and the replication set, indicating substantial heterogeneity. This heterogeneity is likely to be caused by differences in genetic ancestry as most of the replication studies were from the West-African origin, while the population GIGA BioMe included a substantial proportion of persons from East Africa. Differences in LD pattern between causal variants and identified markers as shown in Supplementary Fig. 7 can cause this directionally inconsistent association across studies more commonly known as the flip-flop phenomenon (Lin et al. 2007).

In conclusion, we conducted the first GWAS of POAG comprising continental Africans. We verified the European glaucoma gene *TXNRD2* and identified a novel candidate locus encompassing *EXOC4* that requires further follow up in large African studies. A GRS combining the effects of the known POAG SNPs indicated that these SNPs are implicated to play a role in African POAG. Future studies on POAG in Africa should take the substantial genetic heterogeneity into account by ascertaining large discovery and replication sets from the same geographic area.

## Methods

### Study population

The Genetics In Glaucoma patients from African descent study (GIGA) is a multicenter case–control study comprising POAG patients and controls from South Africa and Tanzania. Participants from Black African and South African Coloured ancestry were ascertained from the ophthalmology outpatient department of the Groote Schuur Hospital in Cape Town, South Africa (*N*_cases_ = 327; *N*_controls_= 194), and from hospitals in Tanzania: Muhimbili National Hospital and CCBRT Disability Hospital in Dar es Salaam (*N*_cases_ = 395; *N*_controls_ = 382). The study was conducted according to the guidelines for human research by the National Institute for Medical Research in Tanzania. Ethical approval was obtained from the institutional review boards at each study site, and written informed consent was provided by each participant.

The Charles Bronfman Institute for Personalized Medicine BioMe BioBank is an electronic medical record (EMR)-linked clinical care biobank that integrates research data and clinical care information of patients at The Mount Sinai Medical Center New York. This center serves diverse local communities of upper Manhattan with broad health disparities including POAG. The current analysis includes participants who self-reported to be of African ancestry (*N*_cases_ = 450; *N*_controls_= 1350) who were enrolled between September 2007 and October 2014. Ethical approval was obtained from the institutional review boards at Mount Sinai, and written informed consent was obtained from all participants.

### Phenotype definition

In GIGA, POAG cases met category 1 or 2 of the ISGEO classification for open-angle glaucoma (Foster et al. 2002). In brief, cases had either a definite visual field defect and Vertical Cup Disc Ratio (VCDR) ≥ 0.7, or VCDR > 0.8 in the absence of a visual field test. Other inclusion criteria were an open angle on gonioscopy and age of onset older than 35 years. Glaucoma patients diagnosed with secondary causes were excluded from this study. Controls were recruited at the same ophthalmology clinics and underwent identical examinations as the POAG cases. Inclusion criteria were: no signs of glaucoma, IOP ≤ 21 mmHg; VCDR< 0.5, and a VCDR inter-eye asymmetry < 0.2, no family history of glaucoma, and age > 55 years. Case and control status was adjudicated by two experienced ophthalmologists (AT and HL).

In BioMe information on POAG status, sex, age was derived from patients’ EMR. POAG patients were considered cases if they had ≥ 1 diagnoses for POAG (ICD-9 codes 365.01, 365.05, 365.11, 365.12 or ICD-10 code H40.11). Participants with pre-glaucoma (ICD-9 code 365), ocular hypertension only (ICD-9 code 365.04), unspecified glaucoma (ICD-9 code 365.10) or with secondary glaucoma (Supplementary Table 8) were excluded from the analyses. Details of the ICD-9 or ICD-10 codes used can be found in Supplementary Table 9. Controls were those of African ancestry over 40 years of age not being diagnosed with any type of glaucoma.

### Genotyping

In GIGA, 1162 participants were genotyped using either the Illumina HumanOmniExpressExome Beadchip (964,193 variants; Illumina, Inc., San Diego, CA, USA; *n* = 999) or the Illumina HumanOmni2.5Exome Beadchip (2,406,945 variants; Illumina, Inc., San Diego, CA, USA; *n* = 163). Extensive quality control (QC) was performed on the genotyped data in PLINK v1.07 (Purcell et al. 2007). Variants with a call rate < 95%, as well as variants showing an extreme deviation from Hardy–Weinberg equilibrium (*P* < 1 × 10^−6^), or MAF < 0.01 were excluded. All SNPs were mapped to genome build hg19/GRCh37. Individual level QC was performed by exclusion of individuals with a call rate < 95%, discordant sex in self-report versus genetically determined sex, excess or reduced heterozygosity, relatedness (PI-HAT > 0.25) or duplicative samples based on identity by descent (IBD) sharing calculations. The final dataset consisted of 663 and 476 successfully genotyped POAG cases and controls, respectively.

Participants from BioMe were genotyped on either Illumina HumanOmniExpressExome-8 v1.0 beadchip array or the illumina Multi-Ethnic Genotyping Array (MEGA). As in GIGA, QC was performed following a similar protocol. Exclusion of variants was based on a call rate < 95%, extreme deviation from Hardy–Weinberg equilibrium (*P* < 1 × 10^−5^), or MAF < 0.01. Individual level QC excluded samples with a call rate < 95%, gender mismatches, ethnic outliers, excess or reduced heterozygosity and first degree relatives or duplicates.

### Imputation

Imputation of unknown genetic variation was performed by means of the “cosmopolitan” approach of using all available populations in a reference panel. The 1000 Genomes Project phase III version 5 was used as an imputation reference panel for GIGA (Genomes Project et al. 2015). The pipeline implemented at the Michigan Imputation Server (https://imputationserver.sph.umich.edu) was used for prephasing and imputation (Minimac) of GIGA genotypes (Das et al. 2016). Imputations of BioMe genotypes were carried out with the program IMPUTE using the 1000 Genomes project phase I version 3 as a reference (Genomes Project et al. 2012; Howie et al. 2009).

### Population structure

In GIGA, the population structure was examined by principal-components analysis (PCA) in PLINK v1.9 (Chang et al. 2015); PCA plots for each array and population are displayed in Supplementary Fig. 8. Scree plots were examined to determine the number of principal-components (PC) for adjustment of potential population stratification (shown in Supplementary Fig. 9). The first five PCs were used as covariates for South African samples, the first four for Tanzanian samples.

In BioMe, population structure (Supplementary Fig. 8) was controlled for by means of genetic matching using the first two PCs. Additional matching was performed based on age and sex.

### Replication

The Eyes of Africa Genetic Consortium, the South London POAG case–control cohort and The African Descent and Glaucoma Evaluation Study (ADAGES III) served as replication panels. The Eyes of Africa Genetic consortium is a Pan-African study of genetic determinants of POAG, and comprises studies recruited from Ghana, Nigeria, South Africa and the USA, totaling a sample size of 2320 POAG cases and 2121 controls. The methods of ascertaining POAG cases has been described in detail elsewhere (Liu et al. 2013). In brief, POAG cases met the following inclusion criteria: glaucomatous optic neuropathy (VCDR > 0.7 or notch in the neuroretinal rim), and visual field loss (examined by frequency doubling technology or standard automated perimetry) consistent with optic nerve damage in at least one eye. Controls were participants with no known first-degree relative with glaucoma, IOP less than 21 mmHg in both eyes without treatment, and no evidence of glaucomatous optic neuropathy in either eye. Genotyping of cases and controls was performed on the Illumina OmniExpressExome array. Genotype QC is described in Supplementary Appendix 2.

The South London POAG case–control cohort consists of 378 POAG patients and 217 controls of West African ancestry residing in the United Kingdom. Patients were recruited from glaucoma clinics in South London and included if they had visual field loss in at least one eye attributed to glaucoma by a glaucoma specialist, had a VCDR of more than 0.6, were receiving intraocular-lowering medication (or had previous surgery), and had open drainage angles on gonioscopy. Controls were recruited from other eye clinics and were included if the examining ophthalmologist deemed there was no sign of POAG, had healthy optic discs (VCDR < 0.6), and normal intraocular pressure without any IOP-lowering therapy (< 20 mmHg). The majority of controls did not have formal visual field testing. Genotyping was performed in a single batch using Illumina’s OmniExpress array. Genotype QC has been described in the Supplementary Appendix 2.

The African Descent and Glaucoma Evaluation Study (ADAGESIII) is a large collection of African American POAG patients and healthy controls recruited at five eye centers in the US (La Jolla, California; New York, New York; Birmingham, Alabama; Houston, Texas; Atlanta, Georgia). The methods of recruitment and selection of POAG cases have been described previously (Zangwill et al. 2018). In brief, eligibility for inclusion as a POAG case required glaucomatous visual field damage defined as a pattern standard deviation or glaucoma hemifield test results outside normal limits. If good-quality visual fields were not available glaucomatous optic disc damage defined as evidence of excavation, neuroretinal rim thinning or notching, localized or diffuse retinal nerve fiber layer defect, or an inter-eye asymmetry of the vertical cup-to-disc ratio of more than 0.2 was required. Controls were ascertained at Wake Forest School of Medicine. Details on genotyping and QC are summarized in the Supplementary Appendix 2.

### Statistical analysis

We conducted a three-stage GWAS. Stage 1 was aimed at the discovery and consisted of a meta-analysis on summary data from GIGA and BioMe. Stage 2 included replication of independent and lead SNPs identified at stage 1 reaching a significance level *P* < 1 × 10^−6^. Stage 3 combined all results in an overall meta-analysis.

Genome-wide association testing in the GIGA study assumed an additive genetic model adjusting for sex and age and included the aforementioned PCs of the principal-components analysis. Association analyses were carried out in EPACTS (http://www.sph.umich.edu/csg/kang/epacts/index.html) by means of the Firth bias-corrected likelihood-ratio test (Firth 1993). In BioMe SNPTEST was applied (https://mathgen.stats.ox.ac.uk/genetics_software/snptest/snptest.html) using the frequentist association tests implemented in the program, based on an additive model (Marchini et al. 2007). Cases and controls were matched by age, sex and the first two principal components in a 1:2 case/control ratio. To control for genotype uncertainty, we used the missing data likelihood score test (the score method).

### Stage 1

A centralized filtering was performed on GIGA and BioMe GWAS results prior to the meta-analysis. Summary result files were assessed and filtered for monomorphic SNPs and SNPs with a minor allele frequency < 0.01. SNP’s that failed or had low-quality imputation, i.e. Minimac *r*^2^/SNPTEST INFO < 0.5, were also excluded. The cleaned summary statistics of both studies were then meta-analyzed by means of an inverse variance fixed effects meta-analysis implemented in METAL (Wilier et al. 2010). Summary statistics were corrected using the ‘genomic-control’ option in METAL to eliminate any residual bias. Only variants present in GIGA South Africa, GIGA Tanzania, as well as BioMe were taken for further analysis.

We searched for evidence of replication of the 15 known POAG variants found in European and Asian GWA studies by employing two replication strategies. First, we used the “exact” approach that involves only the lead significant markers. *P* values at each known POAG SNP in our study were examined and a *P* < 0.05 was considered as evidence for statistically significant replication. Next, we analyzed the transferability of SNPs by applying the “local” approach. All SNPs in strong LD (*r*^2^ > 0.8) with the known POAG SNP in the 1000 Genomes European population were analyzed. For evidence of local transferability, *P* values were adjusted for the number of effective SNPs within a locus as determined by the Genetic Type I Error Calculator (2018) (Li et al. 2012).

To identify potential additional independent signals nearby the lead SNP in the meta-analysis of GIGA and BioMe, we conducted a conditional analysis implemented in Genome-wide complex trait analysis (GCTA 2018) software, using the cojo method, which performs conditional and joint analyses with model selection (Yang et al. 2011). The genome-wide meta-analysis summary statistics from the discovery stage were used as the input data. Within the GCTA analysis, MAF was restricted to ≥ 1% and *P* < 1 × 10^−6^. For this analysis, we used the GIGA Tanzania 1000 Genome phase 3 imputed data to calculate LD between pairwise SNPs. SNPs further than 10 Mb apart were assumed to be in LD.

We next applied haplotype association analysis to identify POAG associated haplotypes that harbor the variants identified in the discovery stage with *P* < 1 × 10^−6^. The haplotype association analysis comprised two steps. First, pairwise measures of LD were calculated in Haploview to identify LD blocks (LD) (Gabriel et al. 2002). Second, significant haplotypes were identified using a Chi-squared test implemented in Haploview (Barrett et al. 2005).

### Stage 2

SNPs put forward for replication were first assessed in each replication sample. *P* value thresholds for significance were adjusted for the number of SNPs tested by the Bonferroni method. Results of all five replication studies were subsequently combined in an inverse variance meta-analysis.

### Stage 3

Finally, results from stage 1 and 2 were combined in a transethnic meta-analysis. SNPs showing evidence of effect heterogeneity between studies (*P*het < 0.05) were analyzed using the Han Eskin random-effects model (Han and Eskin 2011). This analysis implemented in METASOFT software increases the power to detect associations under heterogeneity.

### Power analysis

Power analysis was performed using the Power for Genetic Association analyses (PGA) package (Menashe et al. 2008). For replication of known POAG SNPs power analysis showed that for *α* = 0.05/15 tests, (Supplementary Fig. 6 red line) and minor allele frequencies of 0.05, 0.10, 0.25; minimal OR’s of 1.5, 1.35 and 1.25, respectively, could be detected at statistical significance assuming 80% power. For genome-wide analysis in the discovery stage, we had > 80% power given an alpha of 5 × 10^−8^ to detect variants with odds ratios of 1.89 and 3.25 for effect allele frequencies of 0.05 and 0.01, respectively (Supplementary Fig. 6 green line). For validation of SNPs in stage 2, we had > 80% power at an alpha of 0.05/3 independent SNPs = 0.017 to detect loci with odds ratios of 1.29 and 1.7 for effect allele frequencies of 0.05 and 0.01, respectively (Supplementary Fig. 6 line blue line).

### Bioinformatics analysis

Several bioinformatics tools to assess whether SNPs or their linked genetic variants were associated with a putative function that might affect patient outcomes were consulted. HaploReg v4.1 and the RegulomDB v1.1 that include Genotype-Tissue Expression (GTEx) database from the Encyclopedia of DNA Elements (ENCODE) project were used to identify the regulatory potential on candidate functional variants to examine the particular tracks of interest, such as TF-ChIP signals, DNase peaks, DNase footprints and predicted DNA sequence motifs for transcription factors (Boyle et al. 2012; Ward and Kellis 2012). The GTEx data were used to identify the correlations between SNPs and whole-blood-specific gene expression levels. The Ocular Tissue Database (2018) (IOWA) was checked for expression of associated genes in relevant ocular tissue, in which levels of gene expression are indicated as Affymetrix Probe Logarithmic Intensity Error (PLIER) normalized value [with normalization in PLIER as described in Wagner et al. 2013].

### Gene-based tests

We performed a gene-based test in VEGAS2 (Mishra and Macgregor 2015) to confirm known POAG genes and to identify additional loci not reaching genome-wide significance in a single marker-based analysis. VEGAS2 examines the association from all SNPs across a gene and corrects for gene size and LD between SNPs. The 1000 Genomes phase 3 African populations were downloaded from the VEGAS website and used as the reference panel for pairwise LD correlations. SNPs were allocated to one or more autosomal genes using customized gene boundaries of ± 10 kb.

### Genetic risk score

To further evaluate to which extent known POAG SNPs confer risk in our study, a genetic risk score (GRS) was calculated in the GIGA dataset. Fifteen well imputed/genotyped (Minimac *r*^2^ > 0.5) SNPs that were previously reported in large GWAS were used for constructing the GRS. For each individual, a weighted GRS was computed by multiplying the number of effect alleles with the log (OR) reported in the literature. We assessed the association of the GRS with POAG in a logistic regression model adjusting for sex, age and PCs. An estimation of the attributable genetic risk was calculated using the R package “attribrisk”.

## Supplementary Material

1

7

8

9

10

11

12

2

3

4

5

6

## Figures and Tables

**Table 1:** Demographic and clinical characteristics GIGA study and BioMe

Clinical and demographic characteristics	POAG cases	Controls
GIGA	663	476
Median age, years (IQR)	65.0 (18)	65.0 (12)
Female, *n* (%)	281 (42)	266 (56)
Self-reported ethnicity/race, *n* (%)		
South African coloured	179 (27)	96 (20)
African black	484 (73)	380 (80)
Median proportion GAA, % (IQR)		
South African colored	33.86 (37.26)	33.12 (28.18)
African black	97.90 (7.80)	97.28 (7.81)
BioMe	450	1350
Median age, years (IQR)	64.0 (16)	64.0 (16)
Female, *n* (%)	290 (64)	870 (64)
Median proportion GAA, *%* (IQR)	86.84 (12.84)	86.74 (12.99)

*GAA* genetic African ancestry, *IQR* interquartile range, *POAG* primary open-angle glaucoma, *SD* standard deviation

**Table 2:** Lookup of known POAG SNPs in GIGA BioMe

Genomic region	Exact replication				Local replication						
	Lead SNP	A1	OR (95% CI)	*P* value	EUR Proxy variant	Correlated allele	*r*^2^ between Lead SNP and Proxy	OR	*P* value	Number of effec tive SNPs in LD	*P* adjust
*CDC-TGRFB3*	rs1192415	G	1.05 (0.88–1.26)	0.5811	-	-	-	-	-	-	-
*TMCO1*	rs4656461	G	0.98 (0.85–1.12)	0.7305	rs28504591	A	0.99	1.31 (1.07–1.6)	0.0094	8.3	0.07827
*AFAP1*	rs4619890	G	1.08 (0.93–1.25)	0.3018	rs28605661	G	0.99	1.10 (0.95–1.27)	0.2219	2.98	0.66144
*FOXC1*	rs2745572	A	0.98 (0.85–1.14)	0.8033	-	-	-	-	-	-	-
*GMDS*	rs11969985	G	1.05 (0.92–1.21)	0.4471	rs12530211	T	0.98	1.09 (0.94–1.24)	0.2266	10.65	1
*CAV1-CAV2*	rs4236601	A	1.04 (0.89–1.19)	0.6349	rs10270569	T	0.83	1.08 (0.92–1.28)	0.3411	3.69	1
*8q22*	rs284489	G	0.99 (0.87–1.12)	0.8401	rs10106029	G	0.97	1.09 (0.97–1.24)	0.1428	4.69	0.66926
*CDKN2B-AS1*	rs4977756	A	1.06 (0.94–1.19)	0.3473	rs10712703	d	0.84	1.20 (1.04–1.39)	0.0145	6.15	0.08907
*ABCA1*	rs2472493	G	1.11 (0.98–1.25)	0.09345	rs2437812	C	0.90	1.12 (1–1.26)	0.0560	2.17	0.12131
*ARHGEF12*	rs58073046	G	1.19 (0.71–2)	0.5113	-	-	-	-	-	-	-
*ATXN2*	rs7137828	T	0.82 (0.91–1.16)	0.1047	rs7310615	G	0.97	0.79 (0.62–1.01)	0.05864	1.19	0.06968
*SIX6*	rs10483727	T	1.11 (0.9–1.37)	0.3212	rs6573307	G	0.96	1.10 (0.94–1.28)	0.2355	9.26	1
*PMM2*	rs3785176	C	1.08 (0.91–1.3)	0.3746	-	-	-	-	-	-	-
*GAS7*	rs9897123	T	1.04 (0.92–1.16)	0.544	-	-	-	-	-	-	-
*TXNRD2*	rs35934224	T	1.06 (0.93–1.21)	0.3692	rs16984299	C	0.90	0.83 (0.74–0.94)	0.0032	15.63	0.04984

Lead SNP: SNP reported in European or Asian GWAS associated with primary open-angle glaucoma; A1: effect allele reported in European or Asian GWAS; EUR proxy variant: variant in strong LD (*r*^2^ > 0.8 EUR 1000Genomes) with the lead SNP that has a smaller *P* value in GIGA BioMe than the exact replication of the lead SNP. Cells are left empty (−) when no variant in the queried LD region had a smaller *P* value; correlated allele: proxy variant allele correlated with lead SNP A1 allele [LDlink (Machiela and Chanock 2015)]; effective number of SNPs: number of effective SNPs within the queried 500 kb region calculated by Genetic Type 1 Error Calculator using the 1000 Genomes African samples as a reference(Li et al. 2012); *P* adjust: corrected *P* value for number of effective SNPs; d: deletion

*d* deletion

**Table 3:** Association results for the top SNPs in previously unreported regions with *P <* 1 × 10^−6^ in the discovery phase (GIGA + BioMe)

SNP	CHR	POS	Nearest gene	A1	Meta-analysis GIGA BioMe					
					*N*_cases_/*N*_controls_	Frequency A1 cases/controls	OR (95% CI)	*P* value	*I* ^2^	*P*het
rs9475699	6	56302054	*COL21A1-DST*	A	858/1061	0.22/0.17	1.65 (1.37–1.98)	1.25E-07	41.4	0.1632
rs141186647	7	133634202	*EXOC4*	A	1113/1826	0.04/0.06	0.48 (0.37–0.62)	3.75E-08	29.2	0.2271
rs62023880	15	56770871	*MNS1-ZNF280D*	A	1113/1826	0.30/0.26	1.39 (1.22–1.58)	5.12E-07	30.4	0.2192

*SNP* rsID, *CHR* chromosome, *POS* base pair; nearest gene (reference NCBI build37) is given as locus label, *A1* effect allele, *OR* odds ratio on POAG based on allele A1; *Phet P* value for heterogeneity

**Table 4:** Association and meta-analysis of the discovery and replication studies for the top-ranked loci

SNP	CHR	POS	Nearest gene	A1	GIGA BioMe (discovery)				Meta-analysis replication studies						Meta-analysis discovery and replication				
					Frequency A1 cases/controls	*N*_cases_/*N*_controls_	OR	*P* value	Frequency A1 cases/controls	*N*_cases_/*N*_controls_	OR	*P* value	*I* ^2^	*P*het	Frequency A1 cases/controls	OR^a^	*P* value*	*I* ^2^	*P*het
rs9475699	6	56302054	*COL21A1-DST*	A	0.22/0.17	858/1061	1.65 (1.37–1.99)	1.25E-07	0.22/0.22	2698/2338	0.95^a^ (0.81–1.11)	*0.141* *	59.6	0.042	0.22/0.21	1.04 (0.82–1.31)	*3.49E-06*	85.06	3.03E-06
rs141186647	7	133634202	*EXOC4*	A	0.04/0.06	1113/1826	0.48 (0.37–0.62)	3.75E-08	0.04/0.04	4588/4543	0.97 (0.92–1.02)	0.278	0	0.723	0.04/0.05	0.90 (0.71–1.16)	*5.91E-05*	79.86	4.30E-05
rs62023880	15	56770871	*MNS1-ZNF280D*	A	0.30/0.26	1113/1826	1.39 (1.22–1.58)	5.12E-07	0.28/0.26	4588/4543	1.02 (0.997–1.04)	0.086	0	0.575	0.28/0.26	1.08 (0.97–1.21)	*1.17E-04*	76.31	2.97E-04

*SNP* rsID, *CHR* chromosome, *POS* base pair; nearest gene (reference NCBI build37) is given as locus label, *A1* effect allele, *OR* odds ratio on POAG based on allele A1, *Phet P* value for heterogeneity

* *P* value’s in italics represent Han and Eskin’s random effect meta-analysis *P* values

a OR from a random effect meta-analysis
