# Supplementary material for: Genome-wide association study of primary open-angle glaucoma in continental and admixed African populations
Source: Hum Genet. Author manuscript; Available in PMC 2019 Sep 21. (PMC6754628; doi:10.1007/s00439-018-1943-7)
Supplement: 12 [file NIHMS1049407-supplement-12.docx]

Supplementary Figures

**Genome-wide association study of primary open-angle glaucoma in continental and admixed African populations**

**Pieter W.M. Bonnemaijer^1,2,3,^; Adriana I. Iglesias^1,2,4^; Girish N. Nadkarni^5,6^; Anna J. Sanyiwa^7^; Hassan G. Hassan^8^; Colin Cook^9^; GIGA Study group (for members Appendix 1) Mark Simcoe^10^; Kent D. Taylor^11^; Claudia Schurmann^5^; Gillian M. Belbin^5,12^; Eimear E. Kenny^5,12,13,14^; Erwin P. Bottinger^5^; Suzanne van de Laar^15^;Susan E. I. Wiliams^16^; Stephen K. Akafo^17^; Adeyinka O. Ashaye^18^; Linda M. Zangwill^19^; Christopher A. Girkin^20^; Maggie C.Y. Ng^21^; Jerome I. Rotter^11^;Robert N. Weinreb^19^; Zheng Li^22^; R. Rand Allingham^23^; Eyes of Africa Genetics consortium (for members Appendix 1); Abhishek Nag^10^; Pirro G. Hysi^10^;Magda A. Meester-Smoor^1,2^; Janey L. Wiggs^24^; NEIGHBORHOOD consortium (for members Appendix 1); Michael A. Hauser^23,25^; Christopher J. Hammond^10^;Hans G. Lemij^26^;Ruth J.F. Loos^5,27^; Cornelia M. van Duijn^2^; Alberta A.H.J. Thiadens^1,2^;Caroline C.W. Klaver^1,2,28^**

^1^ Department of Ophthalmology Erasmus MC, Rotterdam, The Netherlands
^2^ Department of Epidemiology Erasmus MC, Rotterdam, The Netherlands
^3^ The Rotterdam Eye Hospital, Rotterdam, The Netherlands
^4^ Department of Clinical genetics Erasmus MC, Rotterdam, The Netherlands
^5^ The Charles Bronfman Institute for Personalized Medicine, Icahn School of Medicine at Mount Sinai, New York, New York, USA
^6^ Division of Nephrology, Department of Medicine, Icahn School of Medicine at Mount Sinai, New York, USA
^7^ Department of Ophthalmology Muhibili University of Health and Allied Sciences/ Muhimbili National Hospital, Dar es Salaam, Tanzania
^8^ Department of Ophthalmology, Comprehensive Community Based Rehabilitation in Tanzania (CCBRT) Hospital, Dar Es Salaam, Tanzania
^9^ Division of Ophthalmology, University of Cape Town, Cape Town, South Africa
^10^ Department of Twin Research and Genetic Epidemiology, King's College London, London, United Kingdom
^11^ Department of Pediatrics, The Institute for Translational Genomics and Population Sciences, Los Angeles Biomedical Research Institute at Harbor-UCLA Medical Center, Torrance, California, USA ^12^ Genetic and Genomic Sciences, Icahn School of Medicine at Mount Sinai, New York, New York, USA
^13^ The Center for Statistical Genetics, Icahn School of Medicine at Mount Sinai, New York, New York, USA
^14^ The Institute for Genomics and Multiscale Biology, Icahn School of Medicine at Mount Sinai, New York, New York, USA.
^15^ Department of Ophthalmology, University Medical Center Utrecht, The Netherlands
^16^ Division of Ophthalmology, Department of Neurosciences, University of the Witwatersrand, Johannesburg, South Africa
^17^ Unit of Ophthalmology, Department of Surgery, University of Ghana School of Medicine and Dentistry, Accra, Ghana
^18^ Department of Ophthalmology, College of Medicine, University of Ibadan, Ibadan, Nigeria
^19^ Department of Ophthalmology, Hamilton Glaucoma Center, Shiley Eye Institute, University of California San Diego, La Jolla, California, USA
^20^ Department of Ophthalmology, University of Alabama at Birmingham School of Medicine,
Birmingham, Alabama, USA
^21^ Department of Biochemistry, Center for Diabetes Research, Wake Forest School of Medicine, Winston-Salem, North Carolina, USA
^22^ Genome Institute of Singapore, Singapore, Singapore
^23^ Department of Ophthalmology, Duke University, Durham, North Carolina, USA
^24^ Department of Ophthalmology, Harvard Medical School, Boston, Massachusetts, USA
^25^ Department of Medicine Duke University Medical Center, Durham, North Carolina, USA
^26^ Glaucoma Service, The Rotterdam Eye Hospital, Rotterdam, The Netherlands ^27^ The Mindich Child Health and Development Institute, Icahn School of Medicine at Mount Sinai, New York, New York
^28^ Department of Ophthalmology, Radboud University Medical Center, Nijmegen, the Netherlands

Supplementary Fig. 1 Locuszoom plots lookup of 15 known POAG SNPs in GIGA BioMe meta-analysis, plotted against 1000 Genomes LD structure of the population where they were originally found. Purple diamond represents the top SNP from the original study.

| A (EUR LD) | B (EUR LD) | C (EUR LD) |
| --- | --- | --- |
| 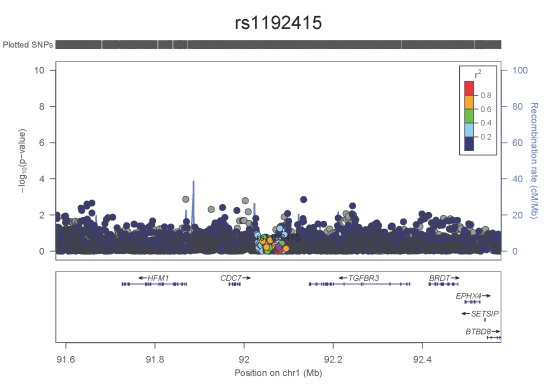 | 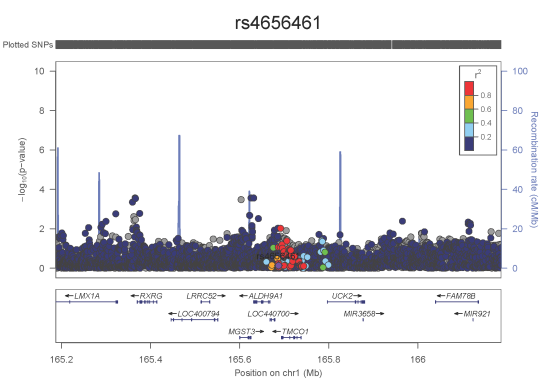 | 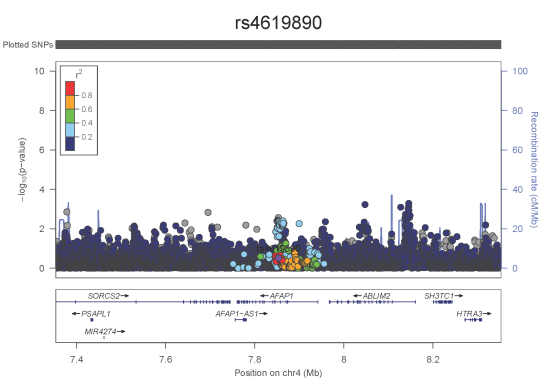 |
| D (EUR LD) | E (EUR LD) | F (EUR LD) |
| 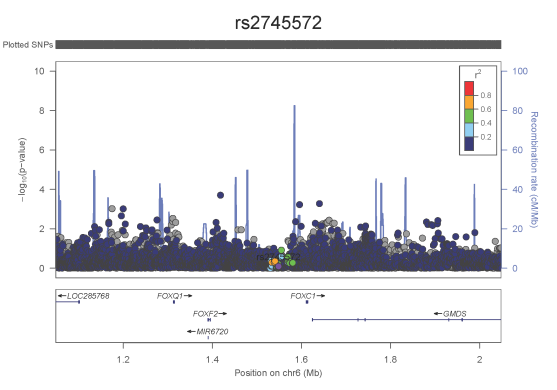 | 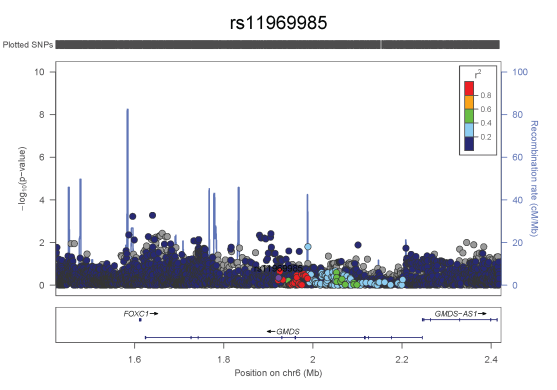 | 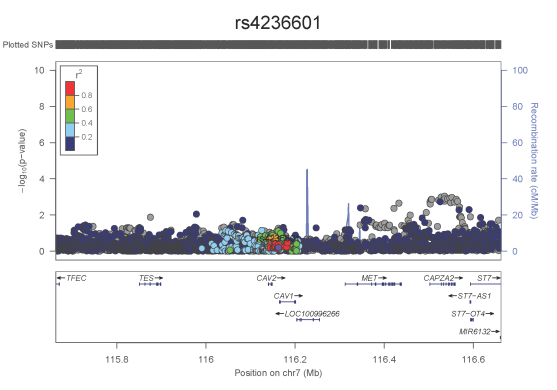 |
| G (EUR LD) | H (EUR LD) | I (EUR LD) |
| 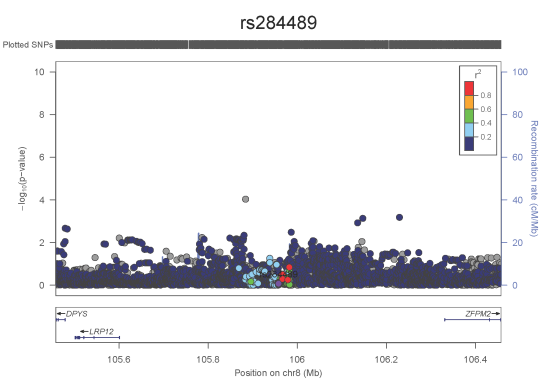 | 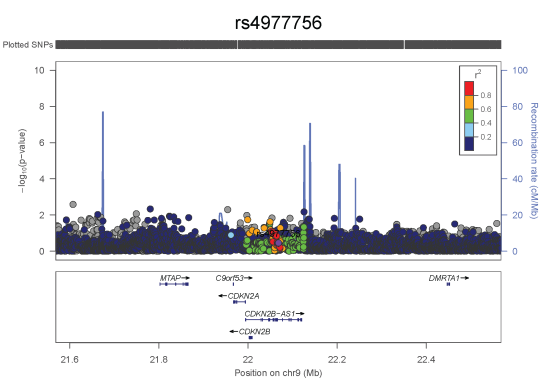 | 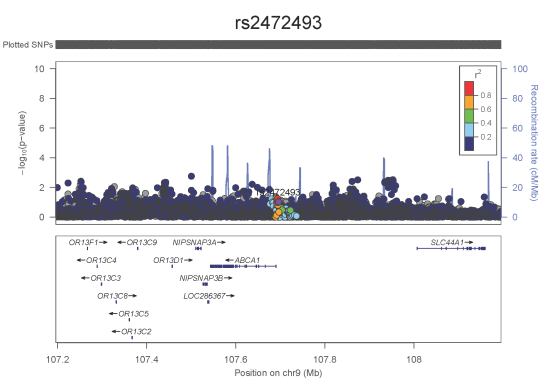 |
| J (EUR LD) | K (EUR LD) | L (EUR LD) |
| 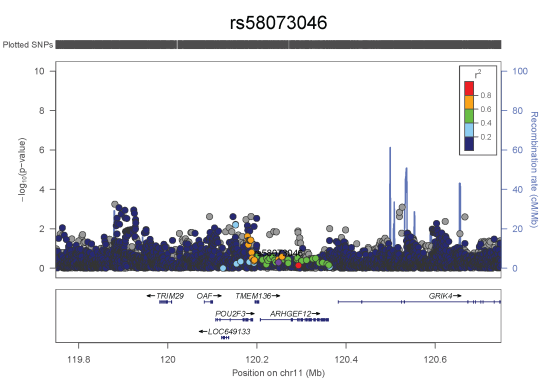 | 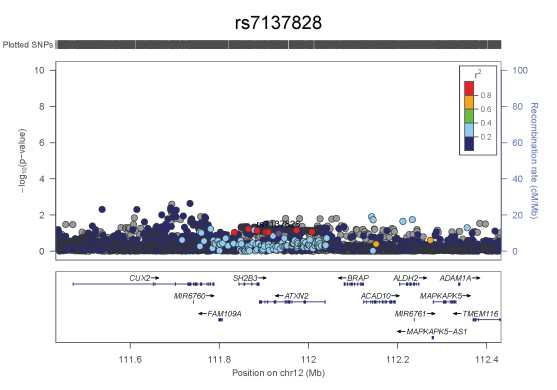 | 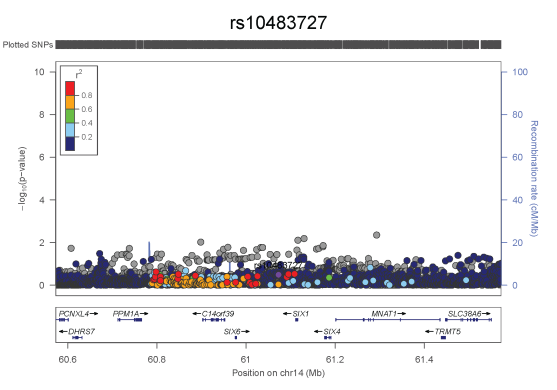 |
| M (ASN LD) | N (EUR LD) | O (EUR LD) |
| 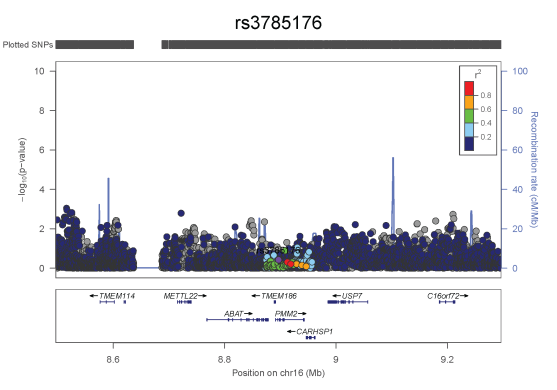 | 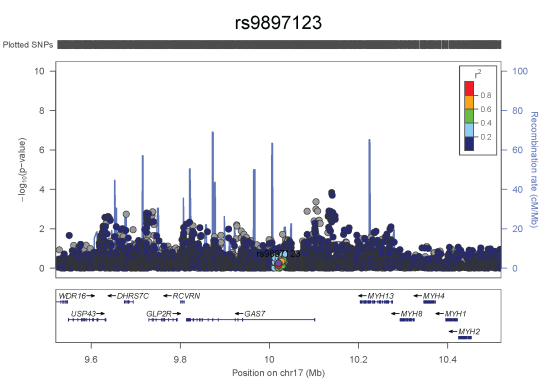 | 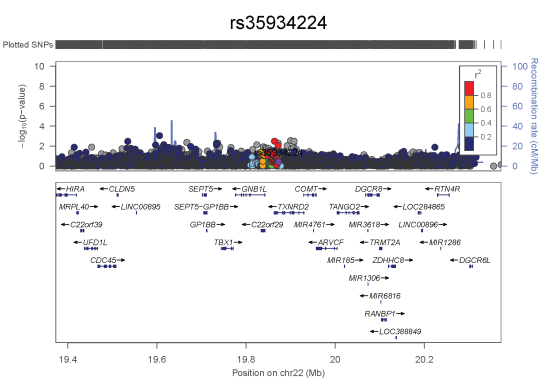 |

Supplementary Fig. 2 Comparison of association effect sizes for 15 known POAG risk SNPs in the original GWAS populations and in GIGA BioMe. Local replication for rs28504591 (*TMCO1*), rs10712703 (*CDKN2B-AS1*), rs16984299 (*TXNRD2*). Errorbars represent the standard error of the association effect in the GIGA BioMe meta-analysis.


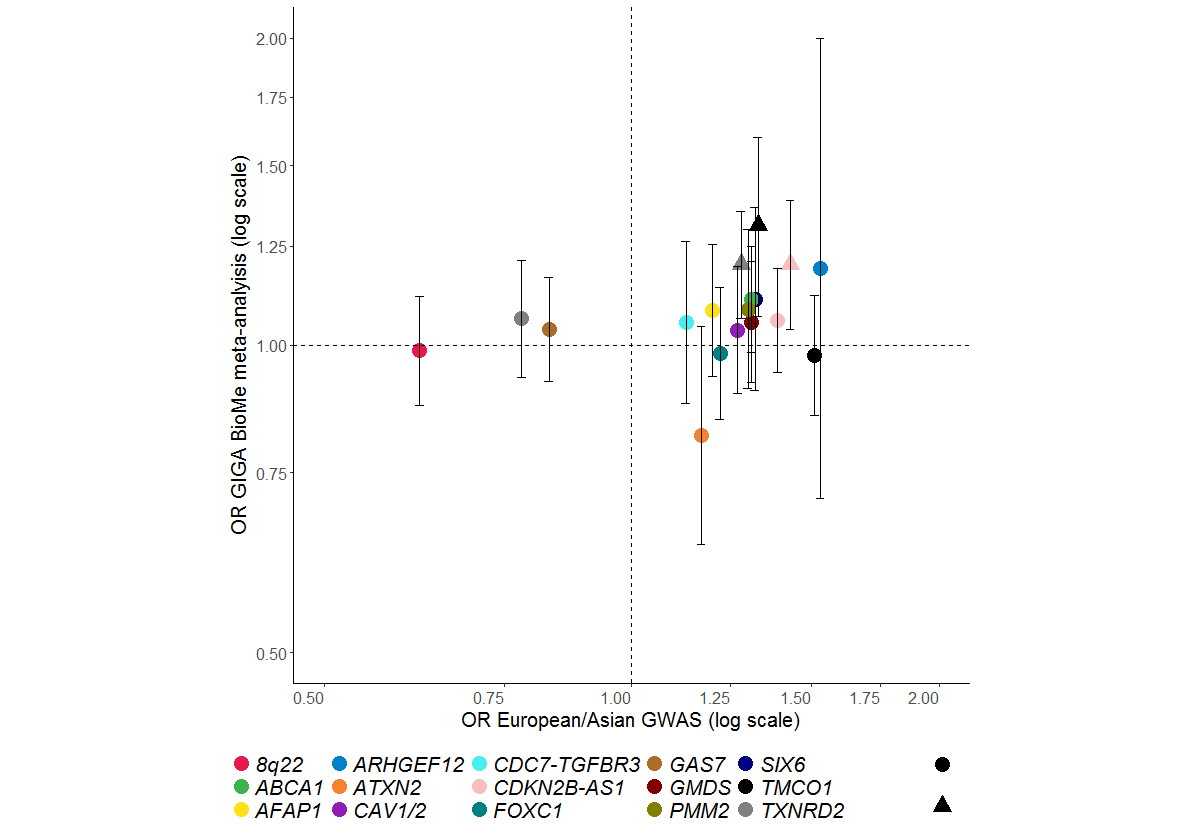


Local replication

Exact replication

Supplementary Fig. 3 Comparison of allele frequencies at 15 known POAG SNPs


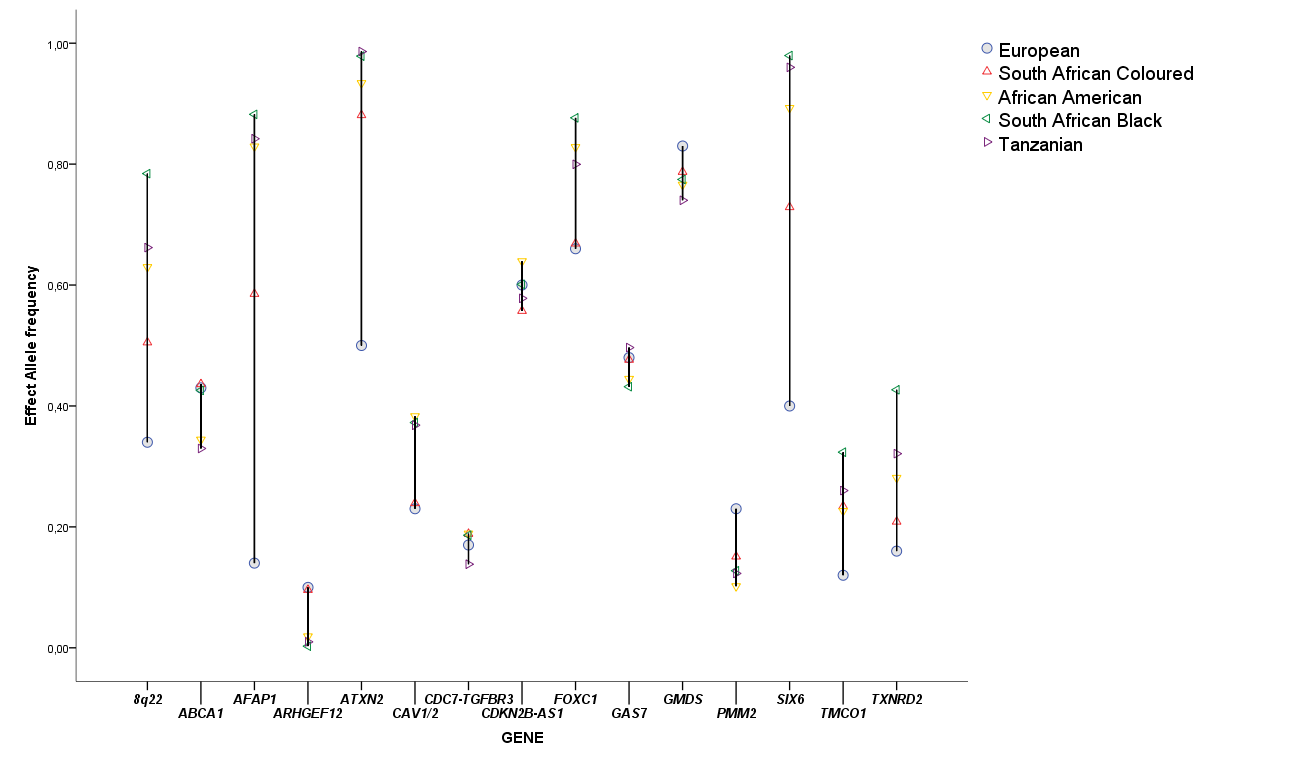


Supplementary Fig. 4 QQ plot discovery phase (GIGA BioMe meta-analysis)


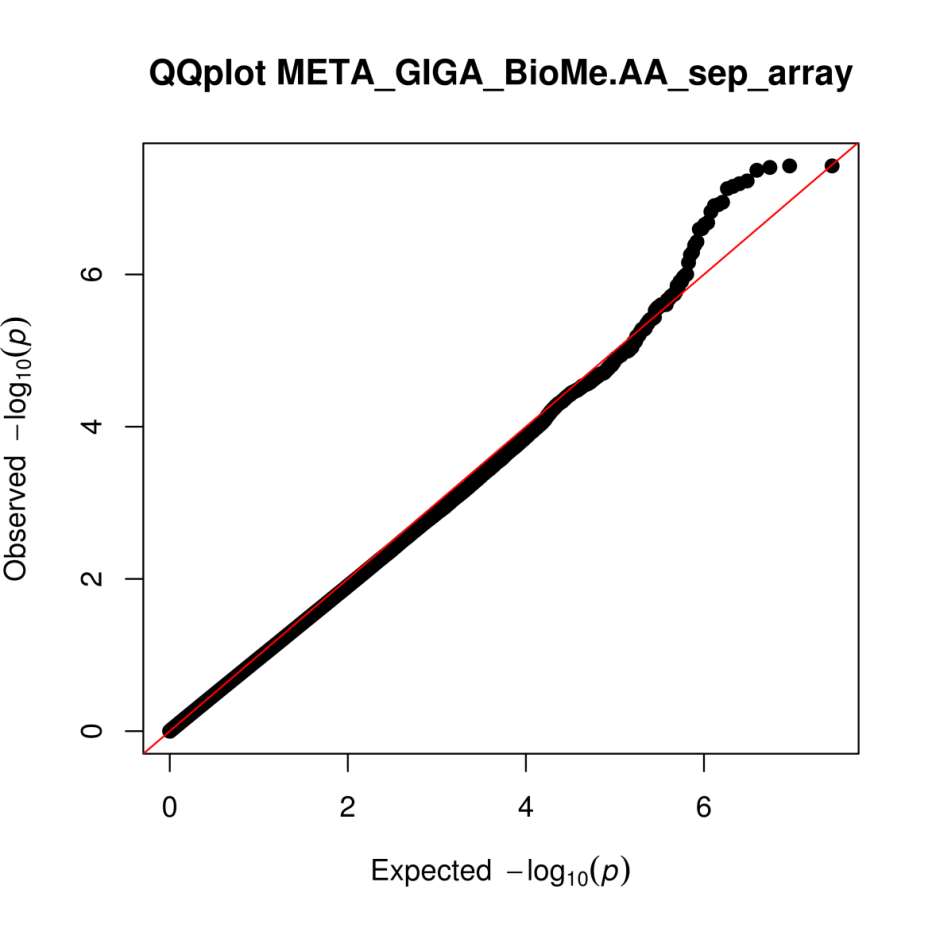


Supplementary Fig. 5 Forest plots meta-analysis discovery and replication stage 3

|  |
| --- |
|  |
|  |

Supplementary Fig. 6 Power Analysis. Graphical representation of the detectable odds ratios(OR) as function of the allele frequency assuming a power of 80%. Red curve presents the detectable OR for validation of 15 known SNPs associated with POAG. Green curve presents the detectable OR to find new significant loci associated with POAG in GIGA BioMe at an α level *P*=5 x 10-8. Blue curve presents the detectable OR for validation of 3 associated loci from the discovery stage in the replication stage α level *P*=0.05/3.

Supplementary Fig. 7 LD plots highlighting differences in LD structure between West African(YRI) and East African (LWK) populations in 1000Genomes

| **1000 Genomes Nigeria (Yoruba) rs9475699 ± 25kb** | **1000 Genomes Kenya Luhya) rs9475699 ± 25kb** |
| --- | --- |
| **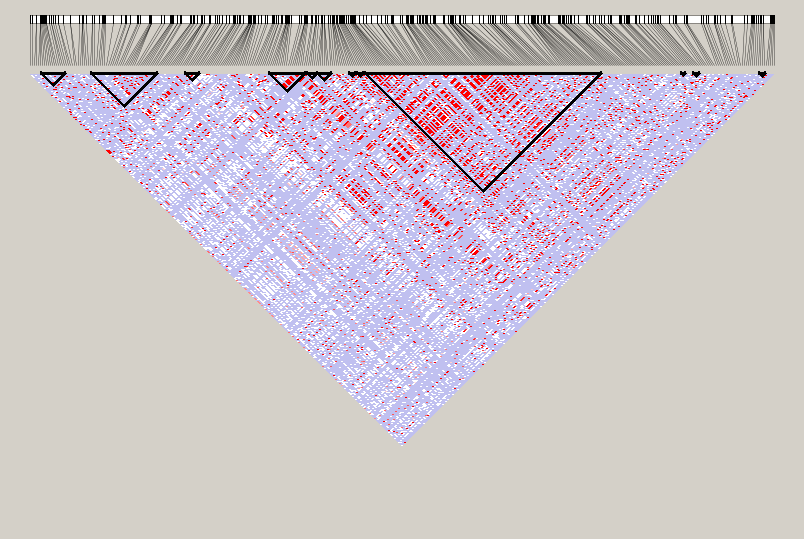** | **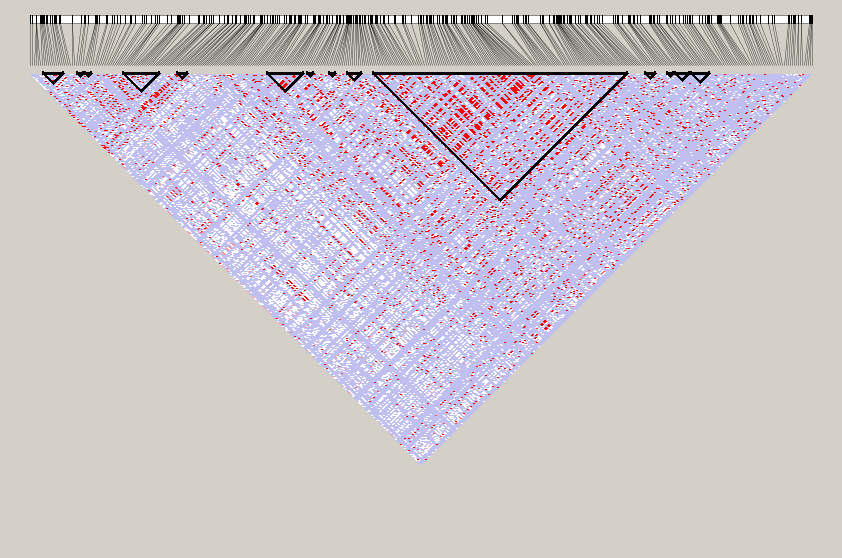** |
| **1000 Genomes Nigeria (Yoruba) rs141186647 ± 25kb** | **1000 Genomes Kenya (Luhya) rs141186647 ± 25kb** |
| **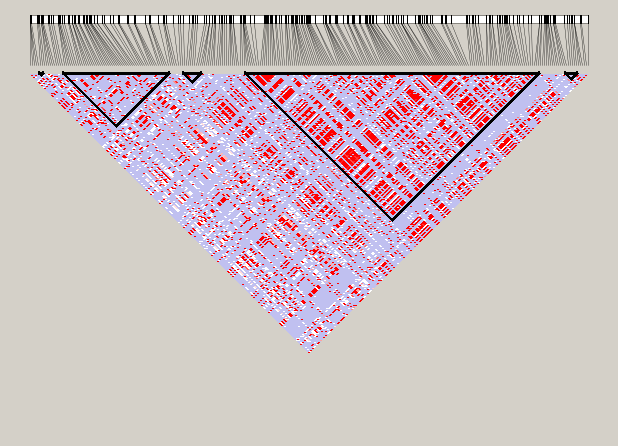** | **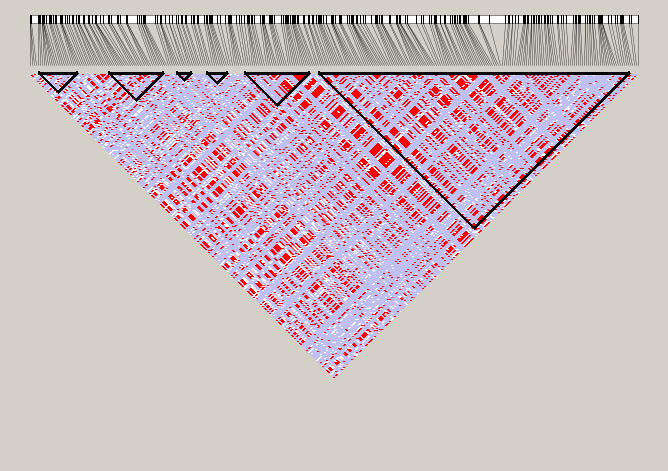** |
| **1000 Genomes Nigeria (Yoruba) rs62023880 ± 25kb** | **1000 Genomes Kenya Luhya) rs62023880 ± 25kb** |
| **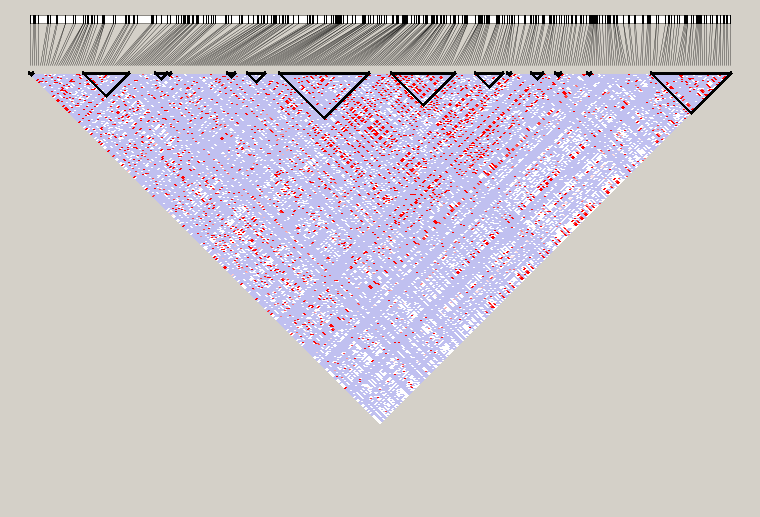** | **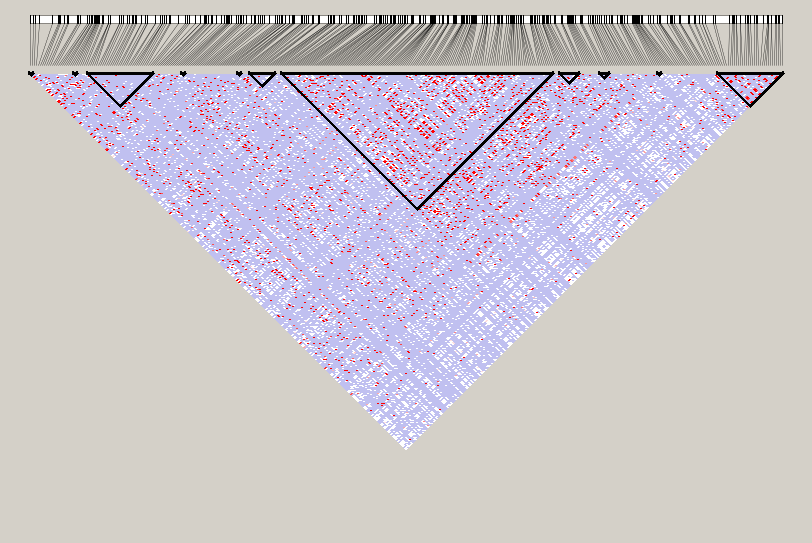** |

Supplementary Fig. 8 Principal component analysis with 1000Genomes

| GIGA study and 1000Genomes Phase3 version 5 |
| --- |
| 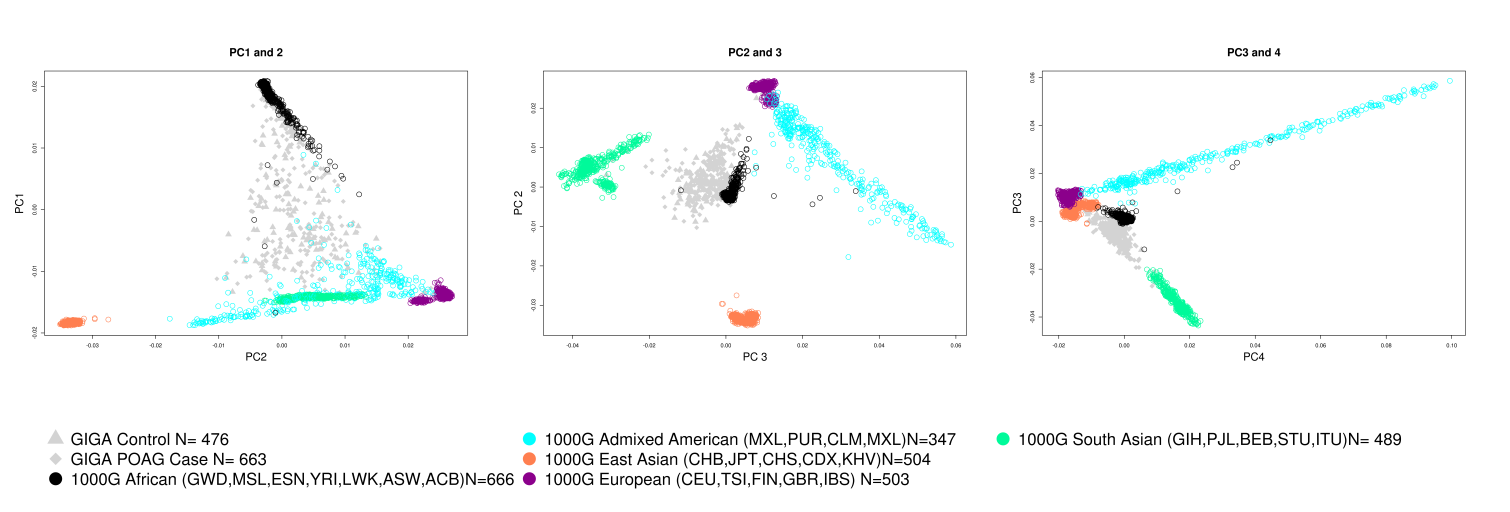 |
| BioMe African American and 1000Genomes Phase1 version 3 |
| 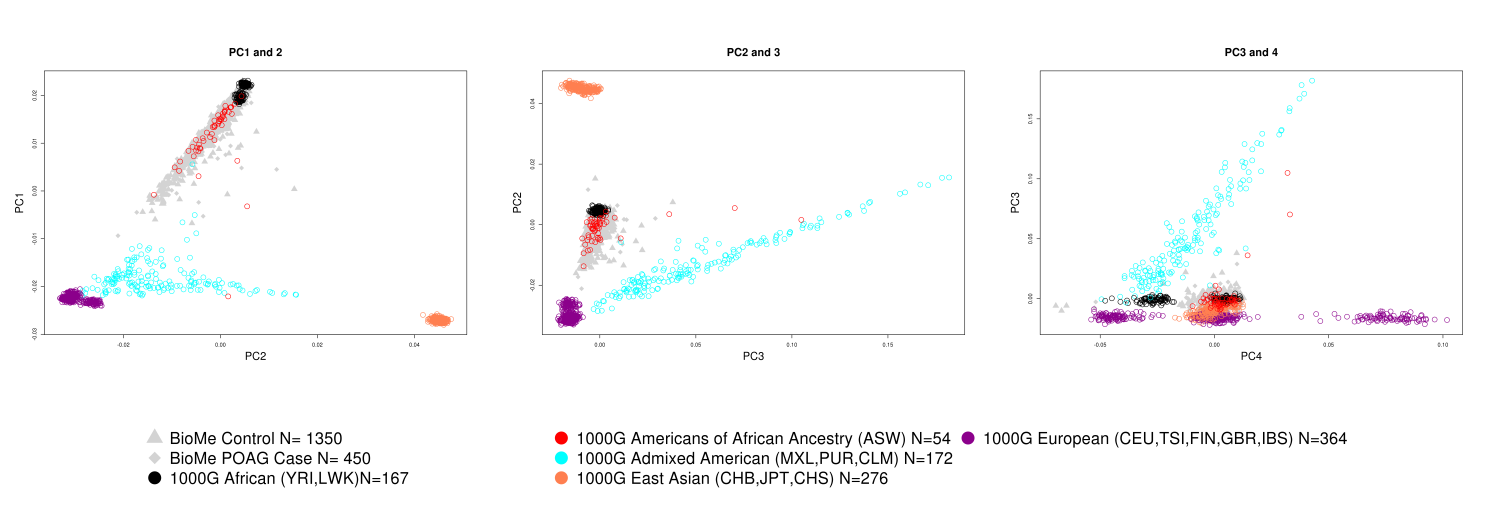 |

Supplementary Fig. 9 Scree plots

| GIGA OMNI2.5 Tanzania | |
| --- | --- |
| 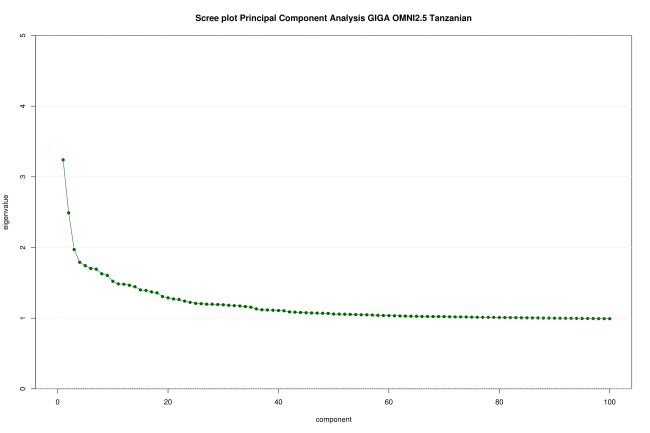 | |
| GIGA OMNI2.5 South Africa | GIGA OMNIEXPRESS South Africa |
| 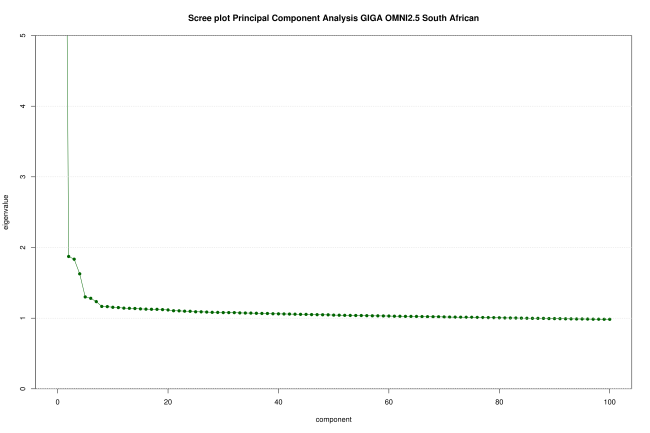 | 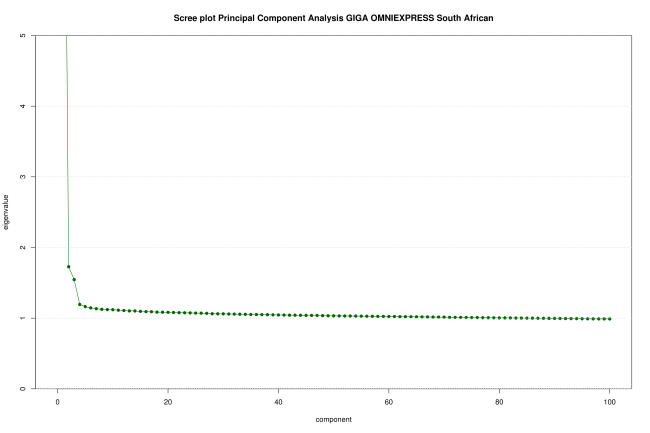 |
| BioMe MEGA African American | BioMe OMNIEXPRESS African American |
| 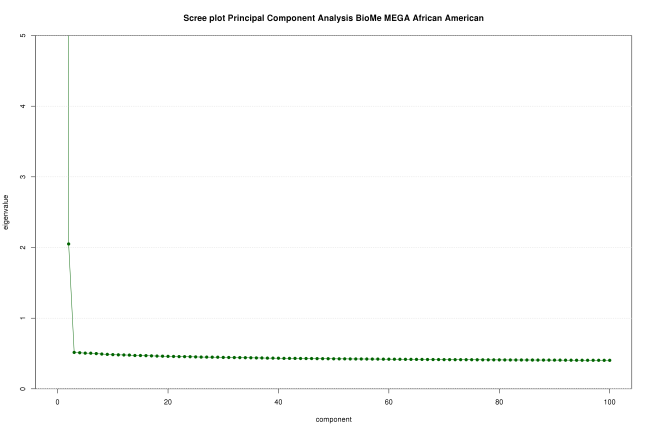 | 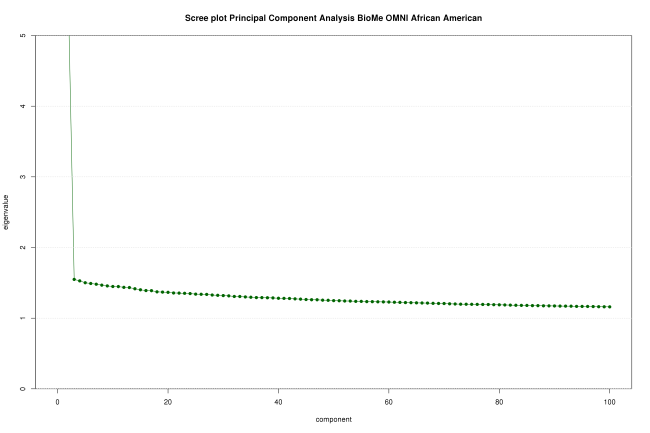 |
